# The riddle of recurrent fever: a clinical approach to pediatric autoinflammatory diseases

**DOI:** 10.3389/fped.2024.1448176

**Published:** 2024-11-15

**Authors:** B. Meertens, L. Hoste, S. J. Tavernier, F. Haerynck

**Affiliations:** ^1^ Department of Pediatric Pulmonology, Infectious Diseases and Immunology, Ghent University Hospital, Ghent, Belgium; ^2^ Primary Immunodeficiency Research Lab, Center for Primary Immunodeficiency Ghent, Jeffrey Modell Diagnosis and Research Center, Ghent University, Ghent, Belgium; ^3^ Center for Medical Genetics, Ghent University Hospital, Ghent, Belgium; ^4^ Department of Biomedical Molecular Biology, Ghent University, Ghent, Belgium; ^5^ Center for Inflammation Research, Laboratory of Molecular Signal Transduction in Inflammation, VIB, Ghent, Belgium

**Keywords:** inborn errors of immunity, monogenic, recurrent fever, periodic fever, autoinflammatory diseases, autoinflammation, inflammasome, pediatric

## Abstract

Autoinflammatory diseases (AIDs) are a group of immunodysregulatory disorders resulting in the increased release or signaling of pro-inflammatory cytokines. Patients with AIDs present systemic inflammation in sterile conditions, which are mainly caused by defects in the innate immune system. Fever is one of the typical symptoms of this derailed immune signaling. In addition, autoinflammatory diseases manifest with varying other symptoms during flare-ups and interphasic periods. The diagnosis of these rare diseases poses numerous challenges. This paper provides an overview of AIDs that arise in childhood and in which fever commonly presents as a symptom. It outlines clinical signs, pathophysiology, diagnosis, and management for each syndrome. Additionally, we discuss a comprehensive diagnostic approach for children where an AID is suspected.

## 1 Introduction

Fever is one of the most common reasons to seek medical care. This is particularly the case for children, as fever makes up 30% of all pediatric ambulatory visits (1). Fever serves as a physiological immune-mediated response to infection, tissue damage or neoplasia. This response leads to inflammation to combat and remove the eliciting agents (2–4). Counter-regulatory signals make that inflammatory episodes ought to be well-controlled. Likewise, immune dysregulation can lead to derailed systemic inflammation and detrimental outcomes. Over 200 clinical conditions are now characterized by persistent or recurrent inflammation and are collectively termed as immune-mediated inflammatory disorders. These disorders can be classified in three main categories: autoinflammatory, autoimmune, and hyperinflammatory diseases (5). AIDs commonly present with signs of systemic inflammation during flare-ups. In addition, subclinical inflammation can be detected during the intercritical phase. While flares typically occur spontaneously, they can be triggered by physical or emotional stress, infection, trauma, hormonal fluctuation, cold, and vaccination. Many autoinflammatory diseases manifest in childhood. They often remain a diagnostic challenge, leading to significant diagnostic delay. Indeed, while underlying diagnoses might be entirely different, presenting symptoms and routine blood examinations can be very similar. This paper will focus on the pediatric autoinflammatory diseases that commonly present with recurrent fever and highlight common and differentiating clinical features between diseases.

### 1.1 Autoimmunity vs. autoinflammation

The first monogenetic defects linked to autoinflammation were described in 1999: familial Mediterranean fever (FMF) and tumor necrosis factor (TNF)-receptor-associated periodic syndrome (TRAPS) (6–8). Autoinflammatory diseases are defined as “clinical disorders caused by defect(s) or dysregulation of the innate immune system, characterized by recurrent or continuous inflammation [elevated acute phase reactants (APR)] and by the lack of a primary pathogenic role of the adaptive immune system (autoreactive T-cells or autoantibody production)”. More than 60 monogenic autoinflammatory syndromes have been described to date (9–11). An overview of the discussed AIDs in this paper is presented in the Supplementary Table 1. In contrast to aberrant activation of the innate immune response in AID, autoimmune diseases reveal a dysregulated adaptive immune response. Paul Ehrlich, who first introduced the concept “autoimmunity”, pointed out the risk of the immune system recognizing self, which he referred to as “horror autotoxicus” (12). The hallmarks of autoimmunity comprise the overactivation of lymphocytes (T and/or B lymphocytes) and the formation of autoantibodies directed against autologous tissue (13). Autoimmune diseases have a female predominance and affect more than 3% of the total population. They can affect a single organ (e.g., diabetes mellitus type I) or they can be systemic (e.g., rheumatoid arthritis (RA) and systemic sclerosis (SSc)). The distinction between dysregulated innate and adaptive immune response, linked to autoinflammation and autoimmunity respectively, serves as a simplified framework for understanding the intricate underlying pathophysiology. Nowadays, this division is considered less clear-cut than previously believed, with overlapping features between both types of inflammatory disorders in various disease states. For example, Systemic Lupus Erythematosus (SLE) was first allocated as a classical autoimmune disease featuring complexes of autoantibodies and self-reactive B- and T lymphocytes, causing arthritis, nephritis and mucocutaneous symptoms. But recent insights in pathophysiology identified complement activation and type 1 interferons as the main drivers of the disease. This indicates that, alongside the adaptive immune system, innate immune signals likewise trigger autoimmune diseases (14). Furthermore, mutations in the NOD-like receptor (NLR), an innate immune sensor forming inflammasome and subsequent activation of IL-1β and IL-18, are strongly linked to AIDs. However, IL-1β also plays a major role in T cell survival and B cell proliferation. These observations highlight the complex interplay between innate and adaptive immunity (15).

### 1.2 Pathophysiology of autoinflammatory diseases

The 2022 International Union of Immunological Societies (IUIS) Phenotypical Classification defines three groups of autoinflammatory disorders, categorized according to their underlying pathophysiology: (1) type I Interferonopathies, (2) defects affecting the Inflammasome and (3) Non-Inflammasome related conditions. An overview of the different AIDs, including their pathophysiology, clinical presentation, and diagnostic work-up, can be found in the Supplementary Table 1. The subsequent sections will delve into each of these subgroups.

#### 1.2.1 Type I interferonopathies

Pathogen recognition receptors (PRRs) in innate immune cells can detect and respond to pathogen associated molecular patterns (PAMPs) or self-derived molecules from damaged cells (DAMPs). PRRs, like toll-like receptors (TLRs) and NOD-like receptors (NLRs), induce downstream inflammatory cascades in an attempt to respond to pathogen invasion and/or damage of cells (16).

**Figure 1:**
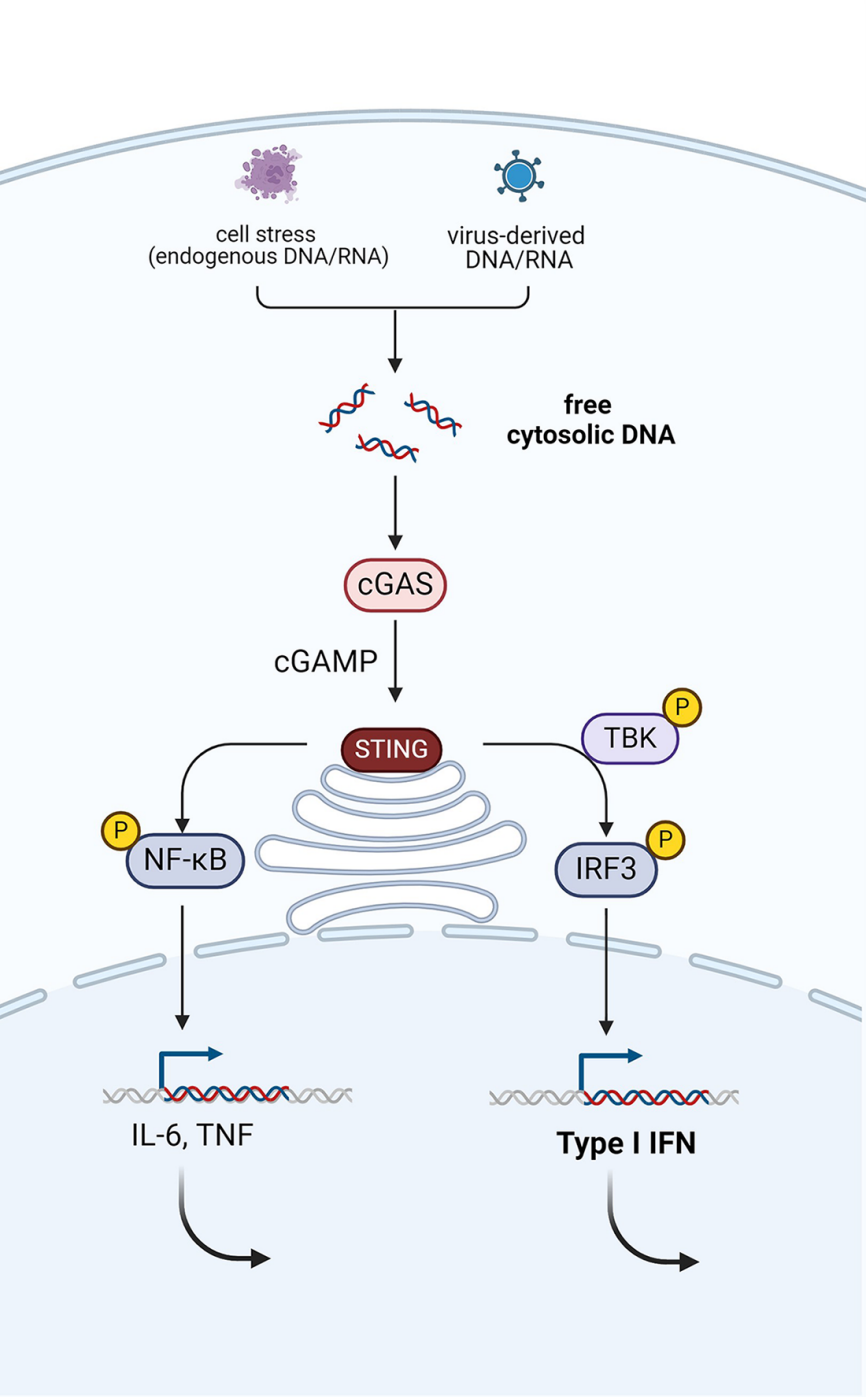
Activation of the cGAS-STING pathway via the detection of pathogenic or endogenous nucleic acids (DNA or RNA). This leads to production of type I interferons via phosphorylation of TANK-binding kinase 1 (TBK1) and interferon regulatory factor 3 (IRF3). Simultaneously, the NF-κB pathway is activated, which mediates the induction of pro-inflammatory cytokines, such as TNF and IL-6. Created with Biorender.com.

PRRs can have a transmembrane localization on the cell surface, like toll-like receptors (TLRs), or can be situated in the cytosol, like RIG-I-like receptors (RLR) or NOD-like receptors (NLRs). An important example of a cytosolic receptor is the cyclic guanosine monophosphate-adenosine monophosphate (cGAMP) synthase (cGAS), which is a central receptor of nucleic acids, derived from pathogenic or endogenous DNA or RNA (see Figure 1) (17).

The messenger of cGAS, cGAMP activates the adaptor protein STING, which results in the production of type 1 interferons (IFNs) (18, 19). The production of type I IFNs in response to recognition of DNA or RNA is an indispensable step in the antiviral response. But inappropriate production of these potent cytokines can have detrimental effects on the host (17). The genetic spectrum of type I interferonopathies extends beyond the cGAS-STING pathway and includes defects in nucleases, innate immune sensors, or adaptor molecules downstream of these sensors and defective feedback of the interferon pathway (20). The common trait among this group of diseases is the upregulation of type I IFN signaling in sterile conditions. As a consequence of the commonalities in the pathophysiological pathways, common clinical signs of different type I interferonopathies include early-onset encephalopathy, brain calcifications—mimicking the sequelae of congenital infections (TORCHES)—and skin vasculitis (exhibiting chilblains). Recurrent fever, however, is not commonly present in patients with type I interferonopathies. Hence, type I interferonopathies will not be discussed in detail, although two prototypical diseases will briefly be mentioned.

Aicardi-Goutières syndrome (AGS) is a prototypical type I interferonopathy (21). Seven different inborn errors of nucleic acid metabolism or sensing have been associated with the clinical phenotype of AGS: *TREX1*, *RNASEH2A*, *-B*, *-C*, *SAMHD1*, *ADAR1* and *IFIH1* (see Supplementary Table 1). The disorder typically presents with neurologic regression during infancy (22, 23). Intracranial calcifications on brain imaging are pathognomonic for AGS. Other neurological symptoms involve epileptic seizures and motor disorders like dystonia, spasticity and paraparesis. More recently, the phenotype of AGS has diversified with many non-neurological features, including chilblains, vasculitis, bowel-inflammation and lupus-like disease (24, 25).

STING-associated vasculopathy with onset in infancy (SAVI) is another monogenic type I Interferonopathy. Due to gain of function mutations in the STING protein, the STING pathway is constitutively activated independently of the production of cGAMP (26). Early onset vasculopathy causes chilblains, distal ischemia of the limbs and arthritis. There is a high mortality rate in this patient group due to interstitial lung disease, evolving to lung fibrosis and respiratory failure (24, 27). To date, no definitive treatment options have been established for these diseases. However, the use of Janus kinase (JAK) inhibitors as immunomodulator in SAVI patients has demonstrated promising outcomes (27).

#### 1.2.2 Defects affecting the inflammasome

Inflammasomes are multiprotein complexes that consist of sensor proteins (or PRRs), apoptosis associated speck-like protein containing a CARD domain (ASC) and protease enzymes such as caspase-1 (28). Upon activation, caspase-1 cleaves pro-IL-1β and pro-IL-18 into their active forms IL-1β and IL-18. Caspase-1 also induces cell death by Gasdermin D (GSDMD)-mediated pore assembly and releases intracellular pro-inflammatory content, called pyroptosis (16, 29).

**Figure 2:**
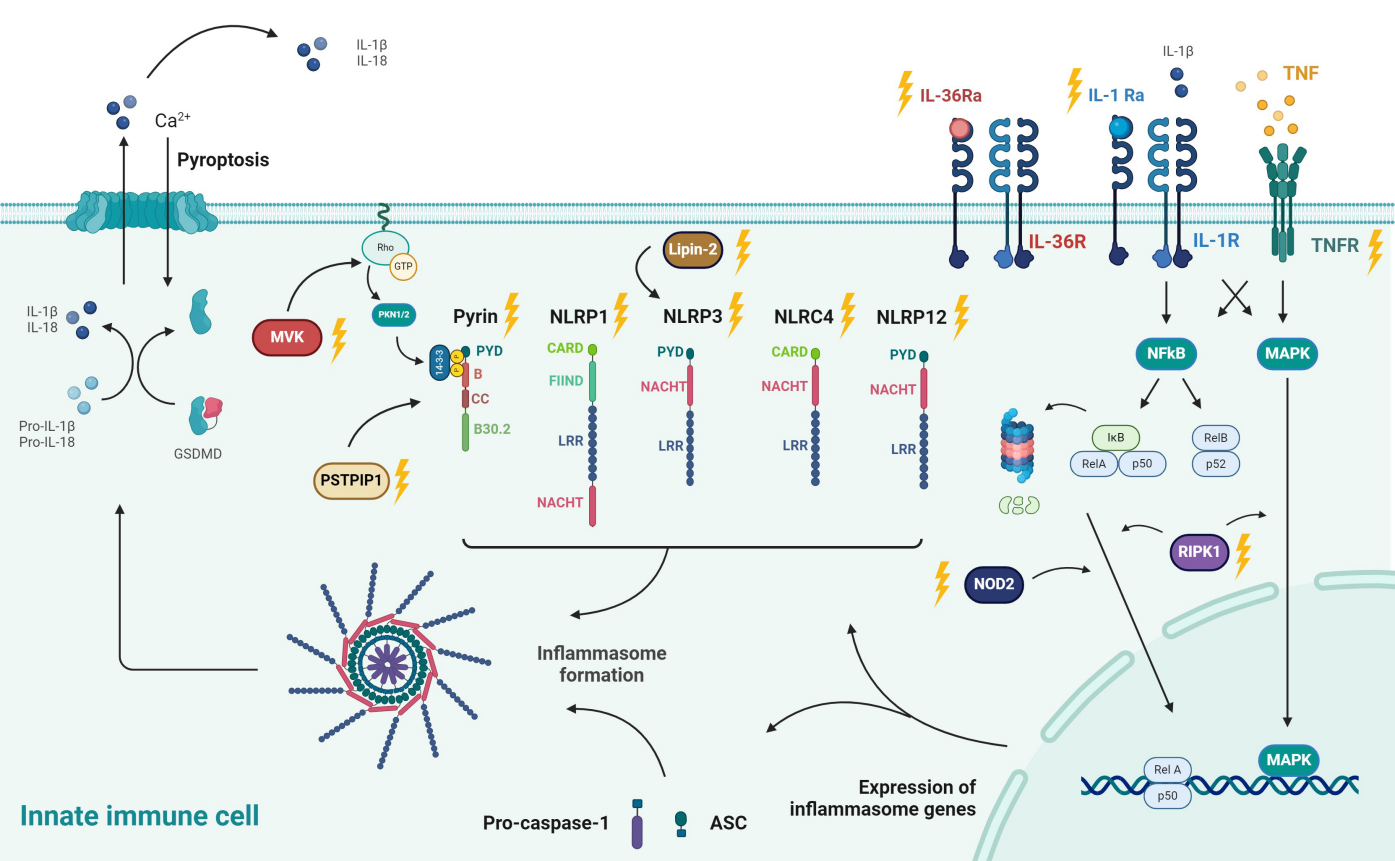
Pathophysiology of autoinflammatory diseases: pathways leading to inflammasome formation and their downstream effects, including pore formation (Gasdermin D; GSDMD), induction of pyroptosis, and secretion of IL-1β and IL-18. Different stimuli for specific inflammasomes (NLRP1, NLRP3, AIM,...) are shown. The proteins associated with heritable autoinflammatory conditions are denoted with yellow lightning bolts. These include MVK (associated with HIDS) and PSTPIP1 (PAPA syndrome) of which the pathways are reported to culminate in derailed Pyrin activity. Pyrin is regulated through Rho-GTPase via PNK1/PNK2. FMF is the most common Pyrin-associated autoinflammatory disease. Besides Pyrin, NLRP1, NLRP3, NLRP12 and NLRC4 are other inflammasomes, respectively associated with NLRP1 deficiency, CAPS, FCAS2, and NLRC4-MAS. Impaired function of Lipin-2, an indirect regulator of NLRP3, leads to Majeed syndrome. Deleterious IL1RN mutations underlie IL-1R antagonist deficiency (DIRA), which is the natural antagonist of the IL-1 receptor. Likewise, mutations in IL36N lead to a defective function of the IL-36 receptor antagonist and cause deficiency of IL-36R antagonist (DITRA). TRAPS is caused by mutations in the TNF receptor (TNFRSF1A) which affects NFκB as a master regulator of transcription of inflammasome-related proteins. Deficiency of RIPK1, a regulator of the NF-κB and MAPK pathways, lead to AIEFL. NOD2 is another protein that activates NFκB and of which deleterious variants underlie Blau syndrome. Created with Biorender.com. Adapted with permission from “Exploring the immune horizon: systemic inflammatory diseases in the era of SARS-CoV-2 and beyond.” by Hoste L. (30). http://hdl.handle.net/1854/LU-01HFQA9EK0NY3S6HEKN4YD1C7P. The PhD thesis of Levi Hoste was published online by Ghent University – Department of Internal Medicine and Pediatrics under the license CC-BY. https://biblio.ugent.be/publication/01HFQA9EK0NY3S6HEKN4YD1C7P.

The family of inflammasome-forming proteins consists of the NOD-like receptor (NLR) proteins NLRP1, NLRP3, NLRP6 and NLR family CARD domain containing 4 (NLRC4), the protein absent in melanoma 2 (AIM2) and pyrin. NLRP1, NLRP3, NLRP6, NLRC4, AIM2 and pyrin undergo oligomerization to form inflammasomes (see Figure 2). Inflammasomopathies are caused by gain-of-function mutations of components involved in inflammasome activation pathways (31, 32). Familial Mediterranean Fever (FMF) is the most common pyrin-associated autoinflammatory disease (PAAD). Pyrin (*Greek for fever*), an intracellular PRR, forms an inflammasome in response to bacterial toxins. Pyrin senses pathogen induced modifications of Rho guanosine triphosphatase (Rho GTPases). The inactivation of RhoA through bacterial toxins causes reduced levels of phosphorylated pyrin via a decreased activity of PKN1 and PKN2 (Figure 2). This in turn releases pyrin of its inhibitory chaperone (14-3-3) protein and induces the formation of an active pyrin inflammasome. Consequently, this leads to the activation and release of IL-1β and IL-18, along with the release of other potent initiators and enhancers of innate immune responses via the process of pyroptosis. Gain-of-function mutations in the *MEFV* gene, which encodes pyrin, cause poor affinity to the regulatory proteins (14-3-3 and PKN1/2) and lead to constitutive activation of the pyrin inflammasome (33, 34). Compared to FMF, mutations in NLRP1, NLRP3 and NLRC4 or their regulatory proteins cause excessive formation and activation of other inflammasomes but equally lead to increases in IL-1b and IL-18 (29).

#### 1.2.3 Non-inflammasome related conditions

The pathophysiology of non-inflammasome related conditions is very heterogenous. The two main and closely intertwined players of this third category of autoinflammatory disorders are TNF and nuclear factor-κB (NF-κB). Activation of TNF family receptors via TNF leads to activation of MAPK and NF-kB pathways. Downstream signaling of NF-kB in turn leads to TNF production (29).

In TNF receptor–associated periodic syndrome (TRAPS), aberrant signaling of the mutant TNF receptor 1 leads to increased activation of MAPK and NF-κB pathways. Furthermore, defective autophagy of TNFR1 also triggers NLRP3 inflammasome activation (35, 36).

Another mechanism of action that calls for a detailed description are the “NFkBopathies” or relopathies, named after the central proteins RelA and RelB in the NF-κB pathway. The NF-kB complex is a central regulator of innate immune activation and is closely linked with the ubiquitination system (37). Activation of NF-κB is complex and can occur via multiple pathways, including the cytosolic NOD2 and the transmembrane IL-1 and TNF receptors. But also, ubiquitination, a post-translational modification process, has an important role in the positive regulation by stabilizing molecular complexes. Activation of NF-κB induces two major signaling pathways: the canonical pathway, which mainly consists of NF-κB1 p50 and RelA, and the non-canonical pathway, which contains NF-κB2 p52 and RelB (see Figure 2). After binding of TNF or IL-1 to their respective cell surface receptors TNFR1 and IL-1R, the canonical NF-κB pathway is activated by proteasomal degradation of the inhibitor of κB (IκB). After release of IκB, p50 and RelA translocate to the nucleus and induce transcription of inflammasome genes (38, 39).

Blau syndrome is an example of a relopathy. It is caused by heterozygous gain-of-function mutations in the NOD2 receptor, and characterized by arthritis, uveitis, and granulomatous dermatitis.

Upstream of the NF-κB pathway, deficiency of the IL-1-receptor antagonist (DIRA) leads to IL-1 hyperactivity and decreased NF-κB inhibition. The same mechanism can be observed in deficiency of the IL-36-receptor antagonist (DITRA), a member of the IL-1 family. Beyond signs of systemic inflammation, disorders of TNF/NF-κB activity exhibit a very diverse phenotype: oral and genital ulcers in haploinsufficiency of A20 (HA20), periorbital edema in TRAPS and granuloma formation in Blau syndrome. Since TNF plays a central role in pathophysiology, TNF inhibitors are the mainstay of the current therapy (13).

#### 1.2.4 Undefined or non-Mendelian AID

Besides the monogenic AIDs outlined earlier, a significant number of patients exhibit non-Mendelian AID. They resemble AID but they lack any known monogenic defect so far. Examples are Behçet disease, systemic juvenile idiopathic arthritis (sJIA), periodic fever with aphthous stomatitis, pharyngitis, and adenitis syndrome (PFAPA), and syndrome of undifferentiated recurrent fever (SURF) (17, 33). PFAPA and SURF will be discussed in section 2.3.

## 2 Overview of pediatric autoinflammatory diseases

In this section, we will discuss autoinflammatory diseases that have an onset during childhood and typically manifest with recurrent episodes of fever. The systemic inflammation during flare-ups can manifest in many different organ systems, such as the serosa (pleural, pericardial or peritoneal), joints, skin or eyes. Our aim is to provide an overview and comparison of the characteristics of these diseases (see Tables 1, 2).

**Table 1:** Overview of the demographic and main characteristics of recurrent fever syndromes.

Class	Disease	Typical age of onset	Sex ratio% (M/F)	Duration of episode	Time between episodes	Triggers
Defects affecting the inflammasome	FMF	1–7 years	60/40	1–3 days	2–4 weeks	Unspecific
	MKD	0–1 year	50/50	3–7 days	2–8 weeks, irregular, decreasing with age	Vaccination
	CAPS	0.1–5 years	50/50			
	NOMID	0–1 month		Chronically	Chronically	No trigger identified
	FCAS	0–5 years		<24 h	Chronically with diurnal pattern	Cold exposure
	MWS	0–5 years		1–3 days		No trigger identified
	NLRC4-MAS	1–2 month	50/50	>7 days	Variable	No trigger identified
	AIADK	1–50 years	55/45	2–10 days	3–6 weeks	Cold exposure
	AIADK	4–5 month	50/50	3–4 days		Unknown
	AIEFL	0–3 month		1–7 days	2–4 weeks	Unknown
Non-inflammasome related conditions	TRAPS	0–13 years	50/50	5–25 days	4 weeks	Unspecific
	PAPA	1–16 years	50/50	Variable		Minor trauma
	Majeed sy	1–2 years	50/50	1–4 days	2–4 weeks	No trigger identified
	DIRA	0–1 month	50/50	Chronically		Unknown
	DITRA	0–60 year	45/55	Chronically		Unspecific
Undef	PFAPA	0.5–5 year	60/40	2–7 days	2–8 weeks with “clockwork regularity”	No trigger identified
	SURF	1–35 years	40/60	3–5 days	4–6 weeks	No trigger identified

Undef, undefined; SURF, syndrome of undifferentiated recurrent fever.

**Table 2:** Overview of the clinical characteristics of recurrent fever syndromes.

	Disease	Oromucosal	Lnn	HSM	Serositis	Skin	Musculosketal	Gastro-intestinal	Eye	FTT	Others
Defects affecting the inflammasome	FMF	No	No	SM	Peritonitis, pleuritis, pericarditis	Erysipelas like rash	Monoarhritis, myalgia	Abdominal pain	Keratoconus	No	Vasculitis (HSP, PAN)
	MKD	Oral/genital ulcers, pharyngitis	Yes	HSM	No	Maculopapular rash	Arhtritis, arhtralgia, myalgia	Abdominal pain, diarrhea, vomiting	RP, cataract, conjunctivitis	No	
	CAPS										
	NOMID	No	Yes	No	No	Urticaria	Arthralgia, myalgia	No	OA, keratitis, uveitis, conjunctivitis	Yes	DD, seizures, SNHL
	FCAS									No	
	MWS									No	SNHL
	NLRC4 MAS	No		SM	No	Maculopapular, urticarial rashes	Arthralgia, myalgia	Abdominal pain, diarrhea	No	Yes	
	FCAS2	No	Yes	SM	No	Urticaria	Arthralgia, myalgia	Abdominal pain	Conjunctivitis	No	Headache, SNHL
	AIADK	No	No	HSM	No	Dyskeratosis	Oligoarthritis	No	No	Yes	
	AIEFL	Oral ulcers, tonsillitis	Yes	HSM	No	No	Arthralgia	Abdominal pain	No		Headache, hallucination
Non-inflammasome related conditions	TRAPS	No	Yes	yes	Peritonitis, pleuritis	Maculopapular, migratory rash	Arhralgia, myalgia, fasciitis	Abdominal pain, vomiting, diarrhea	conjunctivitis, PE	No	
	PAPA	No	No	No	No	Cystic acne, pyoderma gangrenosum	Arthritis	No	No	No	
	Majeed sy	No	No	HSM	No	Neutrophilic dermatosis	Bone pain and limb swelling	No	No	Yes	CRMO, flexion contractures
	DIRA	No	No	HSM	No	Pustulosis and pathergy	Bone pain and limb swelling	No	No	Yes	CRMO, skeletal abnormalities
	DITRA	No	No	No	No	Pustular psoriasis, acrodermatitis	No	No	No	No	
Undef	PFAPA	Pharyngitis, aphtous stomatitis	Yes	No	No	No	No	No	No	No	
	SURF	Oral ulcers, pharyngitis	Yes	HSM	No	Erythema	Arthralgia, arthritis, myalgia	Abdominal pain, vomiting, diarrhea	Periorbital edema, corneal erythema	No	Headache, malaise

CRMO, chronic recurrent multifocal osteomyelitis; DD, developmental delay; FTT, failure to thrive HM hepatomegaly HSM hepatosplenomegaly; HSP, Henoch Schönlein purpura; Lnn, lymphadenopathies; PAN, poly-arteritis nodosa; PE, periorbital edema; RP, retinitis pigmentose; SM, splenomegaly; SNHL, sensorineural hearing loss; SURF, syndrome of undifferentiated recurrent fever; Undef, undefined.

### 2.1 Defects affecting the inflammasome

#### 2.1.1 Familial Mediterranean fever (FMF)

Familial Mediterranean Fever (FMF) is usually caused by homozygous or compound heterozygous mutations in the *MEFV* gene, with 30% of cases showing an autosomal dominant inheritance pattern with incomplete penetrance (34). The *MEFV* gene encodes a protein called pyrin, which is mainly expressed in myeloid cells. As mentioned in section 1.2.2., gain-of-function of the pyrin inflammasome leads to increased IL-1 and IL-18 production, which in turn leads to systemic inflammation. The disease mainly affects people from the Mediterranean basin of Jewish, Turkish, Armenian or Arabic ancestry. However, the Mediterranean epidemiology of the disease is changing due to massive migration, making it more common in Europe, Japan, and North America (40). The prevalence in the general population is 1–5/10,000. In 90% of patients, disease manifestations start before the age of 20 (41). The typical inflammatory attack lasts less than 3 days and can be accompanied by high-grade fever, monoarthritis and serositis, causing abdominal pain (peritonitis) or thoracal pain (pleuritis or rarer pericarditis). However, children often present with subfebrile temperatures or no increase in temperature at all during the episodes (42). It has been reported that many FMF patients underwent a laparotomy or appendectomy because of severe abdominal pain mistakenly interpreted as an acute abdomen (43). Erysipelas-like erythema located on the lower extremities is a very common characteristic of FMF attacks. Furthermore, FMF is associated with a higher risk of vasculitis, including Henoch Schönlein purpura (HSP) and poly-arteritis nodosa (PAN) (40).

During attacks, an elevation of acute phase reactants in the blood (serum amyloid A (SAA), C-reactive protein (CRP) and leukocytosis) can be detected (40, 42). Urine analysis is usually normal but can show proteinuria (42%) or hematuria (13%) (44). The Tel-Hashomer diagnostic classification stipulates that 2 major criteria (recurrent fever and serositis, favorable response of colchicine, amyloidosis AA) or 1 major and 2 minor criteria (recurrent attacks of fever, erysipelas-like erythema, first-degree relative with FMF) must be present for diagnosis (45). For a diagnosis of FMF in children, the presence of two out of the following five criteria is sufficient: fever, abdominal pain, chest pain, arthritis, and a family history of FMF (46). These Yalçınkaya-Ozen criteria have been shown to be more sensitive for the pediatric population (47).

Daily colchicine is the mainstay of FMF management, usually resulting in complete remission or notable reduction in the severity and frequency of the attacks. In case of colchicine-resistance or intolerance, IL-1 inhibitors offer an effective add-on therapy. According to the European Alliance of Associations for Rheumatology (EULAR) the goal of the treatment of FMF is to reach complete control of unprovoked attacks and to minimize subclinical inflammation in between flares. During attacks, non-steroidal anti-inflammatory drugs (NSAIDs) can relieve symptoms. Colchicine has also been proven to be efficient in preventing renal amyloidosis, a life-threatening long-term complication of FMF (48).

#### 2.1.2 Mevalonate kinase deficiency (MKD)

Mevalonate kinase deficiency (MKD) is a very rare autosomal recessive disease caused by mutations in the *MVK* gene. This gene encodes mevalonate kinase, mutations cause a decreased function of the enzyme. In the autoinflammatory disease, Hyper IgD Syndrome (HIDS), approximately 10% residual activity ought to be preserved, whereas in the rarer and more severe metabolic phenotype, mevalonic aciduria (MA), enzyme activity is less than 1%. Because of the scope of this review, we will focus on HIDS.

The majority of HIDS patients are of European ancestry. Disease flares are accompanied by a variety of symptoms. Almost all patients suffer from cervical lymphadenopathy and gastrointestinal complaints such as abdominal pain, diarrhea and vomiting. Mucocutaneous symptoms include aphthous ulcers (60%), maculopapular rash (40%) and pharyngitis (30%). Other frequent manifestations are arthritis or arthralgia of the large peripheral joints (70%), myalgia (40%), splenomegaly (30%) and eye inflammation (15%). In a few cases, an association with macrophage activation syndrome (MAS) has been observed (49, 50). The classification criteria as formulated by the EULAR are based on the confirmatory MVK genotype and include at least one among the following: gastrointestinal symptoms, cervical lymphadenitis or aphthous stomatitis (51). These classification criteria are used to differentiate similar AIDs, but are not validated for diagnostic use. Historically, increased serum IgD was part of the diagnostic criteria for MKD. Because of the lack in sensitivity and specificity, the use of serum IgD as a diagnostic marker is no longer encouraged (49, 52). An alternative diagnostic test is the measurement of urinary mevalonic acid during disease flares (50). Jeyaratnam and colleagues established that urinary increase of mevalonic acid had a 92% sensitivity and 90% specificity in their retrospective cohort. They conclude that MKD appears unlikely in patients with normal mevalonic acid excretion, but it cannot be entirely ruled out (11). Therapeutic management is focused on targeting the downstream cytokine pathways by blocking IL-1 signaling with biologicals, such as anakinra, an IL-1 receptor antagonist and canakinumab, a monoclonal antibody against IL-1β (49, 53).

#### 2.1.3 Cryopyrin-associated periodic syndrome (CAPS)

Cryopyrin-Associated Periodic Syndrome (CAPS), now referred to as NLRP3-associated autoinflammatory disease (NLRP3-AID), was historically described in terms of three distinct clinical entities: Familial Cold Autoinflammatory Syndrome (FCAS), Muckle-Wells Syndrome (MWS) and Chronic Infantile Neurologic Cutaneous Articular syndrome (CINCA), alternatively called Neonatal Onset Multisystem Inflammatory Disease (NOMID). These different presentations of cryopyrin or NLRP3-inflammasomopathies represent a clinical spectrum with multiple shared features but increasing severity (11). Their combined prevalence is estimated at around 1/360,000 (54). NLRP3-AID is caused by heterozygous, gain-of-function mutations in the *NLRP3* gene and results in the overactivation of the cryopyrin inflammasome. NLRP3-AID generally present with low-grade fever, urticarial rash and conjunctivitis. Shared symptoms furthermore include lymphadenopathy, arthralgia and myalgia. Fever is a common clinical sign, but as an isolated feature is usually less debilitating to patients, because measured body temperature does not always reach 38.3°C. Daily symptoms of flu-like malaise and chronic severe fatigue are reported by patients affected by FCAS and MWS. Although symptoms among patients can differ, they tend to be consistent in the same individual (55). Common complications of MWS and NOMID are neurosensorial hearing loss and AA amyloidosis (30%). Central nervous system symptoms, such as developmental delay and seizures, seen in many CINCA/NOMID patients, are a result of chronic sterile meningitis and elevated intracranial pressure (54, 56). There is a clear influence of the circadian rhythm with worsening of symptoms in the afternoon and evening. Generalized cold exposure is a pathognomonic trigger of complaints in FCAS. The diagnosis of NLRP3-AID can be made if raised acute phase reactants (CRP/SAA) can be detected in combination with at least two of the following clinical symptoms: urticarial-like rash (neutrophilic dermatitis), cold/stress-triggered episodes, sensorineural hearing loss, musculoskeletal symptoms (arthritis, arthralgia or myalgia), chronic aseptic meningitis and skeletal abnormalities (epiphyseal overgrowth, frontal bossing), even in absence of genetic confirmation (57). More recently, Gattorno et al. proposed classification criteria to differentiate NLRP3-AID. In the presence of a pathogenic or likely pathogenic *NLRP3* genetic variant, only one of the following symptoms are needed for NLRP3-AID classification: urticarial rash, red eye (conjunctivitis, episcleritis, uveitis) or neurosensorial hearing loss (51). Genetic evaluation is often inconclusive in NLRP3-AID. The presence of variants of uncertain significance (VUS) in the NLRP3 gene, such as V198M, R488K, and Q703K, cannot confirm the diagnosis of NLRP3-AID. These variants, also known as low-penetrance variants, have been described in asymptomatic individuals but may contribute to an NLRP3-AID phenotype in affected carriers, exhibiting both typical and atypical symptoms, such as gastrointestinal symptoms (58). Standard of care for patients with NLRP3-AID consists of IL-1 blockade, and most patients respond well when medication is dosed adequately. In some cases, higher dosages are required for optimal effect (13, 49, 59). Recently, Cosson et al. proposed a new classification system based on the measurement of cytokines release and cell death in a broad range of different mutant *NLRP3* variants. Functional studies revealed a phenotype-genotype correlation with possible specific therapeutic targets in the different groups (60).

#### 2.1.4 NLRC4—macrophage activating syndrome (MAS)

The NLRC4 inflammasome-related pathology is caused by a gain-of-function mutation in the gene encoding for NLR-family CARD domain-containing protein 4 (NLRC4). Patients display recurrent, life-threatening episodes of autoinflammation and infantile enterocolitis (AIFEC) (61, 62). The phenotype of AIFEC includes fever and severe enterocolitis, frequently leading to failure-to-thrive and requiring parenteral nutrition. In some instances, a maculopapular or urticarial rash can be observed. Inflammatory episodes feature macrophage activation and cytotoxic T-cell dysfunction, resembling macrophage activation syndrome (MAS), often presenting early in life. Blood levels of IL-1β and IFNγ are typically upregulated, and an extremely high IL-18 can be measured, differentiating AIFEC from hemophagocytic lymphohistiocytosis (HLH). The trigger for flares is unknown so far. AIFEC can be considered a chronic inflammatory disease. This explains why elevated levels of acute phase reactants (e.g., IL-18) can also be observed in between episodes (63). Treatment with anakinra has been shown to reduce MAS episodes, induce biochemical resolution and lessen the need for systemic corticosteroids (64).

#### 2.1.5 Familial cold autoinflammatory syndrome-2 (FCAS2)

Familial cold autoinflammatory syndrome-2 (FCAS2) is an autosomal dominant disease caused by mutations in the NLRP12 protein, a regulator of NF-κB and caspase-1 activity (65). Besides fever, the most prominent clinical features include urticaria, myalgia, arthritis, abdominal pain and headache. These symptoms are highly similar to the previously described FCAS, an NLRP3-inflammasomopathy. The following features only present in FCAS2, but are rarely observed: sensorineural hearing loss, lymphadenopathies, splenomegaly and thoracic pain. Onset of symptoms in FCAS2 occurs mainly during childhood (70%) and is induced by cold exposure (66). Corticosteroids can shorten fever intensity and duration but are unable to completely control disease manifestations. IL-1 blockade has been shown to be a successful treatment in a few cases (67).

#### 2.1.6 Autoinflammation with arthritis and dyskeratosis (AIADK)

Only three cases have been described in literature of patients with autoinflammation with arthritis and dyskeratosis (AIADK). These patients showed early onset recurrent fever and disseminated dyskeratosis of the skin. During childhood, patients developed oligoarthritis, and signs of autoimmunity with positive antinuclear antibodies (2/3) and autoimmune hemolytic anemia (1/3). Vitamin A deficiency was documented in all patients, although supplementation did not affect disease evolution. Mutations within the *NLRP1* gene have been identified as the etiological basis for the observed clinical phenotype. The exact pathophysiology remains to be unraveled, but increased caspase-1, IL-18 and IL-1β secretion clearly showed enhanced inflammasome activation (68, 69). A good response to anakinra has been reported in one patient (69).

#### 2.1.7 Autoinflammation with episodic fever and lymphadenopathy (AIEFL)

Autoinflammation with episodic fever and lymphadenopathy (AIEFL) is an autosomal dominant disorder caused by RIPK1 deficiency, an important regulator of necroptosis and the NF-κB and MAPK inflammatory pathways. Episodes of high grade fever recur every 2–4 weeks and have an onset in early infancy. The flares are accompanied by painful lymphadenopathy in the cervical, axillary, inguinal, and/or periaortic region. Additional symptoms can include hepatosplenomegaly, tonsillitis and aphthous ulcers. Patients furthermore report headaches, and/or hallucinations that coincided with their fevers. AIEFL shares many characteristics with another clinical syndrome, periodic fever, aphthous stomatitis, pharyngitis, and cervical adenitis (PFAPA), but differs in that AIEFL patients have an earlier age of onset, lymphadenopathy that extends beyond the cervical region, and often experience hepatosplenomegaly or arthralgia (70, 71). Patients showed clinical improvement after treatment initiation of tocilizumab, a monoclonal antibody against the IL-6 receptor (71, 72).

### 2.2 Non-inflammasome related conditions

#### 2.2.1 TNF receptor-associated periodic syndrome (TRAPS)

The TNF receptor-associated periodic syndrome (TRAPS), first named Familial Hibernian Fever (FHF), is an autosomal dominant disease caused by heterozygous, usually missense, mutations in the *TNFRSF1A* gene with poor genotype-phenotype (8). This condition is uncommon, affecting approximately 1 or 2 individuals per million. It typically manifests in childhood median onset at 4.3 years (0–13 years) and affects both sexes equally (73). TRAPS exhibits a range of symptoms, including fever, signs of serositis such as peritonitis and pleuritis, and a migrating maculopapular rash. Although fever is a typical sign, patients exhibit only subfebrile temperatures in 30% of disease flares. Patients typically complain of myalgia located beneath the areas affected by the rash. Eye symptoms may involve conjunctivitis and periorbital edema. Headaches and lymphadenopathies occur less frequently. The duration of disease flares varies greatly between individuals, but tends to be longer compared to other diseases discussed in this paper. This causes a significant disease burden, with an average of 70 symptomatic days per year (74). Many different diagnostic and classification criteria have been proposed. Most recently, Gattorno and colleagues combined molecular and clinical features in their classification criteria, i.e., presence of a confirmatory TNFRSF1A mutation and at least one among the following: duration of episodes ≥7 days, myalgia, migratory rash, periorbital oedema or relatives affected. In the absence of the identification of a pathogenic (or likely pathogenic) variant at least two items need to be present (51).

NSAIDs alleviate symptoms and can be used as supportive medication, but corticosteroids are needed to achieve complete remission of a flare. Unfortunately, the effect of corticosteroids tends to decline over time. Other therapeutic options include IL-1 blockade (canakinumab) and a TNF inhibitor (etanercept) (49, 59, 75).

#### 2.2.2 Pyogenic arthritis, pyoderma gangrenosum and acne (PAPA) syndrome

The Pyogenic Arthritis, Pyoderma gangrenosum and Acne (PAPA) syndrome is an autosomal dominant disorder with incomplete penetrance caused by mutations in *PSTPIP1* (76). Although it has been stipulated that PSTPIP1 directly interacts with pyrin, the exact disease mechanism has not been clarified. The classical clinical triad consists of recurrent episodes of purulent but sterile arthritis, cystic acne and difficult treated pyoderma gangrenosum. Fever occurs inconsistently. The PAPA syndrome has a variable expression; affected patients may show only one or two of the cardinal features. PAPA manifests in patients aged 1 to 16-years-old, featuring recurrent episodes of oligoarticular arthritis, typically triggered by minor trauma and signs of systemic inflammation (77). PASH, characterized by pyoderma gangrenosum, acne, and suppurative hidradenitis, is a related condition (78).

The management of this uncommon AID relies on empirical approaches, tailored to each individual case. High doses of corticosteroids are often inadequate for symptom control, especially to treat skin manifestations. A recent review showed promising outcomes in PAPA patients treated with anakinra and canakinumab (79).

#### 2.2.3 Majeed syndrome

Majeed syndrome is an autosomal recessive autoinflammatory disease caused by mutations in *LPIN2*, which encodes for Lipin-2. Lipin-2 is a regulatory protein and indirectly limits the activation of the NLRP3 inflammasome (80). Patients present with chronic recurrent multifocal osteomyelitis (CRMO), congenital dyserythropoietic anemia, and sometimes neutrophilic dermatosis (81). The recurrent episodes of bone pain caused by CRMO of the long bones and often close to the joints can be accompanied by hepatosplenomegaly in 30% and fever in half of the patients. In severe cases, failure to thrive, growth delay and flexion contractures can occur. The complete blood count shows microcytic anemia and sometimes neutropenia (13%). The severity of the anemia can differ, with around 25% requiring regular erythrocyte transfusions. Patients respond well to IL-1 blockade (82).

#### 2.2.4 Deficiency of the IL-1 receptor antagonist (DIRA)

Deficiency of the IL-1 receptor antagonist (DIRA) is a potentially life-threatening autoinflammatory disease that results from autosomal recessive mutations in het *IL1RN* gene, leading to absence or defective production of the IL-1 receptor antagonist (IL-1RA). This causes unopposed amplification of the proinflammatory effect of IL-1β and IL-1α (83). Affected children exhibit symptoms within the first weeks of life characterized by CRMO, cutaneous pustulosis, and signs of systemic inflammation. Fever may not always be present. The sterile osteomyelitis typically involves the long bones, like in Majeed syndrome, but also vertebrae, ribs and clavicles. The phenotype is variable and can include failure to thrive, hepatosplenomegaly, lung disease, nail changes, diverse skeletal abnormalities (widening of ribs, periosteal reaction, vertebral fusion), eye symptoms (episcleritis, conjunctivitis), vasculitis and venous thrombosis (84). Patients respond extremely well to treatment with anakinra, the synthetic form of IL-1RA) (85).

#### 2.2.5 Deficiency of the IL-36-receptor antagonist (DITRA)

Deficiency of the IL-36-receptor antagonist (DITRA) is an autosomal recessive disorder characterized by episodes of high-grade fever, generalized rash, and disseminated pustules. The pathophysiology of DITRA shows similarities with DIRA (section 2.2.4): biallelic mutations in the *IL36N* gene lead to a defective IL-36 receptor antagonist (IL-36RA), a member of the IL-1 family, and causes uncontrolled action of the proinflammatory IL-36 family members (IL-36α, IL36β and IL36γ) (86). The age of onset varies greatly, from 1 month to 83 years old. The patients typically show generalized pustular psoriasis (GPP) or acrodermatitis continua of Hallopeau (ACH), two subtypes of pustular psoriasis, during the recurrent episodes of systemic inflammation (87).

Management of DITRA is complex, and often involves multiple immunosuppressants. Biologicals such as TNFα, IL-17 and IL-12/23 inhibitors have been shown to be superior to IL-1 inhibitors. Recently, a targeted treatment against the IL-36 receptor (spesolimab) led to promising results in a few cases (88, 89).

### 2.3 Undefined or non-Mendelian AID

#### 2.3.1 Periodic fever with aphthous stomatitis, pharyngitis, and adenitis (PFAPA) syndrome

PFAPA, first described in 1986 by Marshall et al., was initially defined as episodic attacks of fever involving one of the three main symptoms: pharyngitis, cervical adenitis and aphthous stomatitis (90). In regions where FMF is not endemic, PFAPA is considered to be the most common periodic fever syndrome in children. While it was first hypothesized to be a monogenic disease, further research showed that the development of the disease is determined by a complex interplay of polygenetic predisposition and environmental factors. *IL10*, *STAT4*, *CCR1-3* and especially *IL12A* were identified as risk variants for developing PFAPA (91). Serum caspase-1 levels and IL-1 have been found to be increased during disease flares, which is consistent with the presumed inflammasome mediated origin of disease. However, many studies have shown inconsistent or conflicting findings for these and other cytokines (92). The episodes have an onset during the first years of life and usually disappear in adolescence. They typically present with a clockwork regularity every 4–6 weeks (93, 94). During the intercritical period, there is a general well-being with normal growth and development (70). PFAPA is diagnosed through exclusion, as it shares many symptoms with other AIDs. Eurofever/PRINTO clinical criteria require at least seven out of the following eight criteria to be fulfilled for classification: presence of pharyngotonsillitis, 3–6 days’ duration of episodes, cervical lymphadenitis, periodicity and absence of diarrhea, chest pain, skin rash, arthritis (51, 70). Although no monogenic defect was found underlying this AID, heterozygous *MEFV* gene variants have been shown to act as disease modifiers. PFAPA patients with *MEFV* variants present with shorter recurrent fever episodes and a later age of onset (95). An important differential diagnosis to consider is cyclic neutropenia. Because, just like PFAPA, cyclic neutropenia presents with recurrent episodes of fever, aphthous stomatitis, and sometimes cervical adenopathies. Unlike in the case of PFAPA though, the neutrophile counts drop to below 500/µl for 3–5 days.

NSAIDs can be used for symptomatic relief but corticosteroids are the mainstay of treatment during attacks. Because of the rapid effect of a single dose of Prednisolone on the fever, this treatment can be considered as a diagnostic criterium. Only a minority of patients require a second dose. Corticosteroids may have the side effect of increasing the frequency of episodes (96). The interplay between PFAPA and FMF is also evident in their treatment, as colchicine has proven to be effective in reducing the number of attacks in patients with PFAPA. Colchicine is mainly used as prophylaxis in patients with frequent flares. Cimetidine, an H2-receptorantagonist with mild immunomodulating properties, is a second, less frequently used option for prophylaxis (97). The role of tonsillectomy in treating PFAPA remains controversial (91, 98). It has been reported to prevent recurrences in two-thirds of patients, although a recent Cochrane review concluded that the evidence, based on studies with small sample sizes, is of moderate quality. Given the risks associated with surgery, no firm conclusions can be drawn about its place in PFAPA treatment (97, 99, 100). In order to reduce the variation in treatment strategies, the Childhood Arthritis and Rheumatology Research Alliance (CARRA) published the Consensus treatment plan (CTP) for PFAPA in 2020 (100). The CTP defines the four different treatment approaches discussed above: (1) antipyretics, (2) corticosteroids, (3) prophylaxis (colchicine/cimetidine), and (4) surgery. Their aim to use this four-arm CTP to streamline future studies.

#### 2.3.2 Syndrome of undifferentiated recurrent fever (SURF)

A significant number of patients under follow-up for recurrent fever do not fit the profile of the previously described monogenic autoinflammatory diseases, nor do they fulfill the diagnostic criteria of PFAPA syndrome. This heterogenous group is characterized by self-limiting episodes of systemic inflammation with diverse clinical presentations in absence of a confirmed molecular diagnosis and it has been variably referred to as atypical PFAPA, undefined systemic autoinflammatory disease (uSAID), or syndrome of undifferentiated recurrent fever (SURF) (101, 102). In what follows, we will refer to this group as SURF.

The most commonly reported symptoms during disease flares of SURF are fever, malaise, abdominal pain, arthralgia, myalgia and eye manifestations (periorbital edema and/or corneal erythema) (103, 104). Papa and colleagues proposed a preliminary case definition for patients with a clinical suspicion of SURF: they define as mandatory features recurrent fever with elevated inflammatory markers (at least three similar periods in 6 months) and negative PFAPA criteria, and genotype for other SAIDs. Additional supporting features are monthly attacks lasting 3–5 days accompanied by fatigue/malaise, abdominal pain, arthralgia/myalgia, eye manifestations and a favorable response to treatment with colchicine/anti-IL1 (103). A recent study by Vyzgha and colleagues illustrates the diagnostic challenges clinicians face, demonstrating a potential overlap between PFAPA and SURF. Data of patients with PFAPA, SURF and uSAID were analyzed from three international registries (Eurofever, JIRcohort and AID-net). Part of the population diagnosed as SURF (26%–37%) met the criteria for PFAPA. Likewise 8%–17% of PFAPA patients fulfilled the preliminary case definition for SURF. This clinical heterogeneity highlights the complexity of classifying patients with recurrent fevers and underscores the need for updated definitions to differentiate SAIDs, in order to optimize treatment strategies (105).

In contrast to PFAPA, SURF patients will rarely show a favorable response to tonsillectomy or on-demand steroid therapy (105). The majority of SURF patients exhibit a complete and consistent response to colchicine. In more severe or therapy resistant cases, anti-interleukin (IL)-1 treatment (mainly anakinra) is the most effective and frequently used alternative (103, 106).

## 3 Discussion: diagnostic work-up

As for other rare diseases, the diagnostic process of autoinflammatory diseases (AIDs) can be difficult. First and foremost, it is key that autoinflammation is considered in the differential diagnosis of children presenting with fever of unknown origin, particularly in patients with recurrent episodes of systemic inflammation at an early age with atypical associated symptoms. Because the diagnosis relies to a great extent on clinical suspicion, earlier studies have shown that there is a median diagnostic delay of 7.3 years. And, although there has been a noticeable overall trend towards improvement in recent years, patients generally still have to wait for a median time of 5 years before a diagnosis is established (107, 108). Flare-ups significantly impact patients’ quality of life, while chronic subclinical inflammation can also contribute to substantial morbidity and mortality. In most autoinflammatory syndromes an adequate treatment is available to control the severity and frequency of attacks and to prevent long-term sequelae (like AA amyloidosis). For these reasons, it is necessary to improve early recognition and diagnosis in this patient population. Table 3 provides an overview of red flags indicating when to suspect autoinflammation in a pediatric patient.

**Table 3:** Red flags: when to suspect autoinflammation in a pediatric patient?

Red flags
• Recurrent fevers without apparent infectious causes 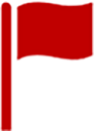 • Periodic fevers with a predictable pattern • Poor response to antibiotics • Mouth or genital ulcers • Lymphadenopathies • Signs of serositis or arthritis • Unexplained rashes, especially in association with fever episodes • Elevated inflammatory markers (e.g., CRP, ESR) during and between episodes • Family history of autoinflammatory disorders • Ethnic background associated with certain autoinflammatory diseases (e.g., Mediterranean descent for Familial Mediterranean Fever) • Young age at the onset of disease flares • Failure to thrive

**Figure 3:**
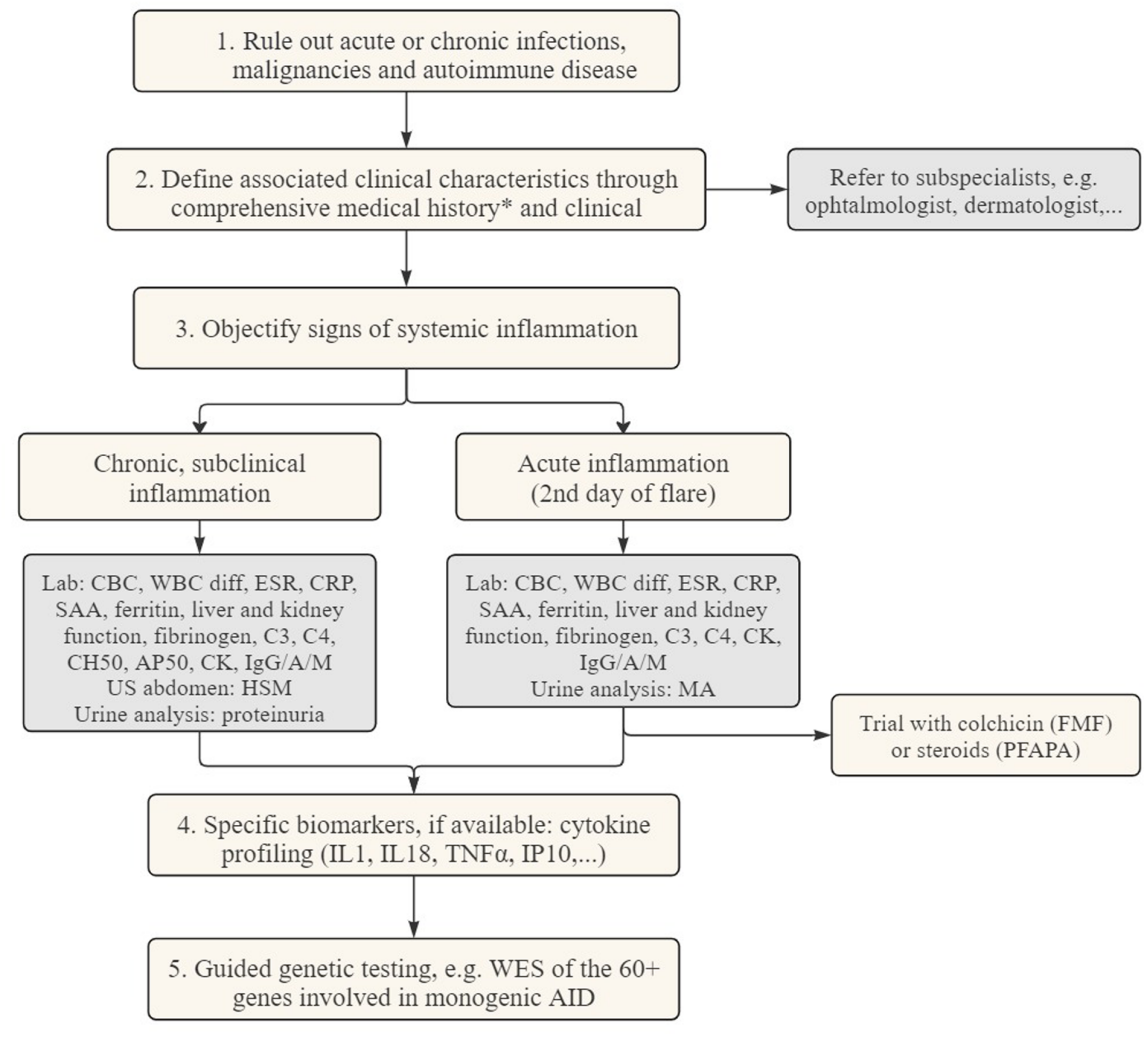
Diagnostic flowchart for patients suspected of autoinflammatory diseases. CBC complete blood count CK creatine kinase CRP c-reactive protein ESR erythrocyte sedimentation rate HSM hepatosplenomegaly MA mevalonic acid SAA serum amyloid A WBC diff white blood cell differentiation WES whole exome sequencing. *Medical history: duration of flares, time between episodes, possible triggers, ethnicity, associated symptoms, failure to thrive.

To guide the clinician in this diagnostic challenge, we designed a diagnostic algorithm (see Figure 3). In a first step, more common causes for systemic inflammation, such as infectious, autoimmune diseases or malignancies have to be ruled out (109). Subsequently, AID episodes (duration, interval) and the accompanying symptoms should be listed in a diary to identify a pattern. In combination with the age of onset and possible triggers of the emergence of fever, the number of potential diagnoses can be narrowed down. An overview of those demographical and clinical parameters can be found in Tables 1, 2. It is necessary to always consider family history and ethnicity. As autoinflammatory diseases present with systemic symptoms, a multidisciplinary work-up is necessary, including consultations with the ophthalmologist to screen for (asymptomatic) uveitis, conjunctivitis, periorbital edema, or retinitis pigmentosa, and the dermatologist to evaluate skin lesions, and if necessary, perform a skin biopsy.

In the following step, documentation of inflammation is needed. To objectively assess systemic inflammation, laboratory measurements can be used during disease flares. These include complete blood count (CBC), white blood cell differentiation and the measurement of acute-phase reactants such as C-reactive protein (CRP), ferritin, serum amyloid A (SAA), and erythrocyte sedimentation rate (ESR), or other biomarkers associated with complement, coagulation, and fibrinolysis systems. Urine analysis can be helpful for the detection of mevalonic acid and repeated urine sampling is necessary during a febrile episode. To evaluate subclinical, chronic inflammation, an assessment is required during the asymptomatic period. This includes repeated blood sampling, evaluation for hepato- and/or splenomegaly by ultrasound, as well as a urine analysis, to screen for proteinuria to detect renal AA amyloidosis.

Immunomodulatory therapy can also serve as a diagnostic criterium, with corticosteroids being administered in suspected cases of PFAPA or colchicine in suspicion of FMF or SURF.

Routine lab tests are usually significantly up- or downregulated during the acute episodes, but they have the disadvantage of lacking specificity. Therefore, comprehensive testing modalities, illustrated in step 4, have been developed in research settings in order to enhance the recognition of underlying diagnoses. One of the methods that holds the promise of making additional distinctions to differentiate the AIDs, where routine lab measures fail to do so, is cytokine profiling. The most common technique to quantify cytokines are enzyme-linked immunosorbent assay (ELISA)-based methods (110). The cytokines that are best known in the context of autoinflammatory diseases are IL-1, IL-18 and TNF (see Figure 2). Although cytokines are signaling molecules that can have many different functions depending on the context, IL-1, IL-18 and TNF are relatively specific markers for autoinflammation. Since the cytokine release in inflammasome activation is one of the key drivers of disease, it can also be a target for therapy and disease monitoring. An example of this targeted therapy can be seen in the management of NLRP3-AID. Indeed, IL-1 blockade, via canakinumab, a monoclonal antibody against IL-1β, and anakinra, a recombinant IL-1 receptor antagonist (IL-1Ra), have proven to be very effective (111). The type I interferonopathies are linked with an increased serum level of IFNα/β, which are also examples of potent pro-inflammatory cytokines. Because the direct detection of type I interferons is difficult to measure, quantification of IP-10 can be a valuable alternative. IFN-γ-inducible protein 10 (IP-10) is both induced by type I and type II interferons. Measuring cytokines can be challenging for different reasons: they are present in low concentrations (range of pg/ml), have short half-lives and are very sensitive to pre-analytical variations. Indirect methods to uncover cytokine signatures have been developed, of which the interferon stimulated genes (ISG) scores is best validated. The ISG score consists of a qPCR test that evaluates the expression of specific genes targeted by type I IFNs. Although ISG score testing may lead to false positives due to viral infections (especially in the pediatric population), experience clearly demonstrates that they are helpful in the further elaboration of type I interferonopathies (19, 112). Sönmez HE and colleagues developed a clinical score that allows clinicians to identify patients with a high suspicion of interferonopathy. These patients may benefit from a further work-up, including the performance of ISG score and cytokine profiling (113).

A final important question to address is the role of genetic testing in the diagnostic work-up. There is a consensus that patients with suggestive clinical symptoms should be genetically investigated (114). Although each single disease has a very low occurrence rate, the diagnostic yield of combined gene panels increases year by year. A study in 2022 by Le Goueff et al. demonstrated a diagnostic rate of 23% with a gene panel consisting of 502 genes (115). Next-generation sequencing (NGS) is now broadly used and has replaced the “gene by gene” method of Sanger sequencing (75). The *Infevers* database is a valuable source of information about all sequenced variants associated with AIDs that can be used by geneticists and clinicians (116). The European Molecular Genetics Quality Network Guidelines put forward a genetic diagnosis of monogenic autoinflammatory diseases in 2020 (117). The interpretation of a genetic variant is full of possible pitfalls due to polymorphisms and incomplete penetrance. Evaluating variants of unknown significance (VUS) and estimating their clinical implications can be challenging. Therefore, it is essential to call upon an experienced team of genetics and clinicians to evaluate the results of genetic testing (114).

## 4 Conclusion

Pediatric autoinflammatory diseases are characterized by recurrent flares of symptomatic systemic inflammation and chronical subclinical inflammation. In this review we summarize the pathophysiology, clinical features, diagnosis and treatment of the most common autoinflammatory diseases with an onset in childhood. We focus in particular on the diagnostic work-up of recurrent fever in children with suspicion of autoinflammation. Even though AIDs are rare, the number of diseases has been rapidly expanding during the past 20 years. By unraveling the pathophysiology of these immune disorders, additional insights can be gained into innate immune regulation and the complex interplay between innate and adaptive immunity, which in turn can improve the treatment of inborn errors of immunity in general. Even in an era of high throughput genetic screening, there is still significant diagnostic delay for patients living with an AID, having major impacts on quality of life and leading to additional morbidity and mortality. After exclusion of infection, autoimmunity and malignancies, it is therefore important to consider autoinflammation in the differential diagnosis of patients presenting with recurrent fever. This overview of AIDs with an onset in childhood can be a useful tool for clinicians and facilitate early diagnosis and treatment of these rare diseases.
