# Supplementary material for: The riddle of recurrent fever: a clinical approach to pediatric autoinflammatory diseases
Source: Front Pediatr. 2024 Nov 15;12:1448176. doi: 10.3389/fped.2024.1448176 (PMC11605516; doi:10.3389/fped.2024.1448176)
Supplement: Supplementary file 1 [file Table1.pdf]

Overview of autoinflammatory diseases

Class	Disease	Genetic defect	Functional defect	Clinical picture	Diagnostic work-up
TYPE 1 INTERFERONOPATHIES	AD STING-associated vasculopathy, infantile-onset	TMEM173 AD	STING activates both the NF-κB and IRF3 transcription pathways to induce expression of IFN	Early-onset inflammatory disease, skin vasculopathy, inflammatory lung disease, systemic autoinflammation and ICC, FCL.	APR, Capillaroscopy, chest CT, PFT, Cardiac US, ANA/ANCA, cytokine profiling (IP-10), ISG score, genetic analysis
	AR STING-associated vasculopathy, infantile-onset	TMEM173 AR		FTT, early onset rash, fever, dyspnea, interstitial lung disease/pneumonitis, polyarthritis, autoAbs, increased inflammatory markers, IFN gene signature.	
	Aicardi-Goutières Syndrome	TREX1 AR-AD (AGS1)	Intracellular accumulation of abnormal ss DNA species leading to increased type I IFN production	Progressive encephalopathy, ICC, cerebral atrophy, HSMG, leukodystrophy, thrombocytopenia, elevated hepatic transaminases, chronic cerebrospinal fluid (CSF) lymphocytosis.	CBC, liver function, APR, X-ray vertebrae and ribs, brain imaging (MRI/CT), EEG, CSF analysis, ophthalmological evaluation, capillaroscopy, cytokine profiling (IP-10), ISG score, genetic analysis
		RNASEH2A AR (AGS2	Intracellular accumulation of abnormal RNA-DNA hybrid species leading to increased type I IFN production		
		RNASEH2B AR (AGS3)			
		RNASEH2C AR (AGS4)			
		SAMHD1 AR (AGS5)	Controls dNTPs in the cytosol, failure of which leads to increased type I IFN production		
		ADAR1 AR (AGS6)	Catalyzes the deamination of adenosine to inosine in dsRNA substrates, failure of which leads to increased type I IFN production		
		IFIH1 AD GOF (AGS7)	IFIH1 gene encodes a cytoplasmic viral RNA receptor that activates type I interferon signaling through the MAVS adaptor molecule		
DEFECTS AFFECTING THE INFLAMMASOME	Familial Mediterranean Fever (FMF)	MEFV AR LOF - AD	Increased inflammasome-mediated induction of IL1β	Recurrent fever, serositis and inflammation responsive to colchicine. Predisposes to vasculitis and inflammatory bowel disease.	APR, urine sediment, genetic analysis, trial with colchicine
	Mevalonate kinase deficicency (Hyper IgD syndrome)	MVK AR	Affecting cholesterol synthesis, pathogenesis of disease unclear	Cervical adenopathy. Oral aphtosis. Diarrhea. Mevalonate aciduria during attacks.	APR, (IgD), urine MA, abd US, cytokine profiling, genetic analysis
	Cryopyrin-Associated Periodic Syndrome (CAPS)<br>Muckle-Wells syndrome	NLRP3 AD GOF	Defect in cryopyrin, involved in leukocyte apoptosis and NF-κB signaling and IL-1 processing	Continuous fever, urticaria, Deafness (SNHL), conjunctivitis, amyloidosis.	APR, audiogram, ophthalmological evaluation, CSF analysis, X-ray joints, urine sediment, cytokine profiling, genetic analysis
	Familial Cold Autoinflammatory Syndrome 1			Non-pruritic urticaria, arthritis, chills, fever and leukocytosis after cold exposure.	
	Neonatal onset multi system inflammatory disease (NOMID) or chronic infantile neurologic cutaneous and			Neonatal onset rash, with continuous fever and inflammation. Aseptic and chronic meningitis, chronic arthropathy. Mental retardation, sensorineural deafness and visual loss in some patients.	

	articular syndrome (CINCA)				
	Familial cold autoinflammatory syndrome 2 (FCAS2)	NLRP12 AD GOF		Non-pruritic urticaria, arthritis, chills, fever and leukocytosis after cold exposure.	APR, audiogram, abd US, cytokine profiling, genetic analysis
	NLRC4-MAS (macrophage activating syndrome)	NRLC4 AD GOF	Gain of function mutation in NLRC4 results in elevated secretion of IL-1β and IL-18 as well as macrophage activation	Severe enterocolitis and macrophage activation syndrome.	APR (ferritin!), enterocoloscopy, cytokine profiling (IL18), genetic analysis
	Autoinflammation with arthritis and dyskeratosis (AIADK)	NLRP1 AR	Systemic elevation of IL-18 and caspase 1, suggesting involvement of NLRP1 inflammasome	Dyskeratosis, autoimmunity and arthritis	CBC, APR, ANA, vit A, cytokine profiling, genetic analysis
	Autoinflammation with episodic fever and lymphadenopathy (AIEFL)	RIPK1 AD	Increased inflammatory markers and pro-inflammatory cytokines/gene signature	Autoinflammatory disorder: regular/prolonged fevers, lymphadenopathy, HSM, ulcers, arthralgia, GI features	APR, abd US, cytokine profiling, genetic analysis
NON-INFLAMMASOME RELATED CONDITIONS	TNF receptor-associated periodic syndrome (TRAPS)	TNFRSF1A AD	Mutations of 55-kD TNF receptor leading to intracellular receptor retention or diminished soluble cytokine receptor available to bind TNF	Recurrent fever, serositis, rash, and ocular or joint inflammation.	APR, urine sediment, cytokine profiling, genetic analysis
	Pyogenic sterile arthritis, pyoderma gangrenosum, acne (PAPA) syndrome	PSTPIP1 (C2BP1) AD	Disordered actin reorganization leading to compromised physiologic signaling during inflammatory response	Destructive arthritis, pyoderma gangrenosum, inflammatory skin rash, myositis, acute-phase response during attacks.	APR, synovial fluid analysis, skin biopsy, cytokine profiling, genetic analysis
	Chronic recurrent multifocal osteomyelitis and congenital dyserythropoietic anemia (Majeed syndrome)	LPIN2 AR	Undefined	Chronic recurrent multifocal osteomyelitis, transfusion-dependent anemia, cutaneous inflammatory disorders.	CBC, APR, MRI limbs/joints, abd US, cytokine profiling, genetic analysis
	Deficiency of the Interleukin 1 Receptor Antagonist (DIRA)	IL1RN AR	Mutations in the IL1 receptor antagonist allow unopposed action of Interleukin 1	Continuous inflammation. Neonatal onset of sterile multifocal osteomyelitis, periostitis and pustulosis.	APR, X-ray/MRI full body, skin/bone biopsy, abd US, chest CT, cytokine profiling, genetic analysis
	Deficiency of interleukin-36 receptor antagonist (DITRA)	IL-36RN AR	Mutations in IL-36RN leads to increase IL-8 production	Life-threatening, multisystemic inflammatory disease characterized by episodic widespread, pustular psoriasis, malaise, and leukocytosis.	APR, skin biopsy, cytokine profiling, genetic analysis
	Blau syndrome	NOD2 (CARD15) AD	Mutations in nucleotide binding site of CARD15, possibly disrupting interactions with lipopoly-saccharides and NF-kB signaling	Uveitis, granulomatous synovitis, camptodactyly, rash and cranial neuropathies, 30% develop Crohn colitis.	APR, skin biopsy, ophthalmological evaluation, cytokine profiling, X-ray joints, calprotectin, genetic analysis
UNDEFINED	Periodic fever with aphthous stomatitis, pharyngitis, and adenitis syndrome (PFAPA)	No known monogenetic defect	Undefined, presumed inflammasome mediated origin	Episodic attacks of fever, pharyngitis, cervical adenitis or aphthous stomatitis	APR, cytokine profiling, trial with corticosteroids
	Syndrome of undifferentiated recurrent fever (SURF)	No known monogenetic defect	Undefined	Self-limiting episodes of systemic inflammation with diverse clinical presentation	APR, cytokine profiling, genetic analysis, trial with colchicine

Supplementary Table 1. Overview of the presented autoinflammatory diseases, adapted from the IUIS classification of Tangye et al, 2022 (10).

Abd US abdominal ultrasound *AD* autosomal dominant *AGS* Aicardi-Goutières syndrome *AIHA* autoimmune hemolytic anemia *APR* acute phase reactants *AR* autosomal recessive *Class* Classification *CBC* complete blood count *CSF* cerebrospinal fuid *FCL* familial chilblain lupus *GOF* gain-of-function *ICC* intracranial calcifcation *HSM* hepatosplenomegaly *IFN* interferon *LOF* loss-of-function *MA* mevalonic acid *PFT* Pulmonary Function Test *SLE* systemic lupus erythematosus *SNHL* sensorineural hearing loss *SP* spastic paraparesis
